# hUCMSC-extracellular vesicles downregulated hepatic stellate cell activation and reduced liver injury in *S. japonicum*-infected mice

**DOI:** 10.1186/s13287-019-1539-8

**Published:** 2020-01-09

**Authors:** Liyang Dong, Yanan Pu, Xiaojun Chen, Xin Qi, Lina Zhang, Lei Xu, Wei Li, Yongbin Ma, Sha Zhou, Jifeng Zhu, Yalin Li, Xuefeng Wang, Chuan Su

**Affiliations:** 1 State Key Lab of Reproductive Medicine, Jiangsu Key Laboratory of Pathogen Biology, Department of Pathogen Biology and Immunology, Center for Global Health, Nanjing Medical University, Nanjing, 211166 Jiangsu People’s Republic of China; 2 Department of Nuclear Medicine, The Affiliated Hospital of Jiangsu University, Zhenjiang, 212000 Jiangsu People’s Republic of China; 3 Department of Central Laboratory, The Affiliated Hospital of Jiangsu University, Zhenjiang, 212000 Jiangsu People’s Republic of China

**Keywords:** Human umbilical cord mesenchymal stem cells, Extracellular vesicles, Schistosomiasis, Liver injury

## Abstract

**Background:**

Accumulating evidence shows that mesenchymal stem cells (MSCs) have the potential as a cellular therapy avenue for schistosome-induced liver injury. Extracellular vesicles (EVs) are membranous vesicles released by almost all cell types, and EVs produced by MSCs (MSC-EVs) exert therapeutic effects in several disease models. However, the potential of MSC-EVs in schistosomiasis treatment remains unclear.

**Methods:**

Using survival analysis, HE and Masson’s trichrome staining, immunohistochemical, western blot analysis, real-time PCR, and EdU proliferation, we investigated the effects of human umbilical cord MSC-derived EVs (hUCMSC-EVs) on the survival and liver injury in the *S. japonicum*-infected mice and explored the underlying mechanism.

**Results:**

Here, we found that like hUCMSCs, hUCMSC-EVs significantly ameliorated liver injury and improved the survival of schistosome-infected mice. Indeed, the hUCMSC-EV-mediated alleviation of liver injury is associated with decreased expression of α-smooth muscle actin (α-SMA), collagen 1, and collagen 3. More importantly, we showed that hUCMSC-EVs directly suppressed the proliferation of LX2 (human hepatic stellate cell) in vitro. In addition, hUCMSC-EVs significantly downregulated the activation of LX2 after transforming growth factor-β1 (TGF-β1) treatment.

**Conclusion:**

Our results provided the first evidence that hUCMSC-EVs reduced liver injury in *S. japonicum*-infected mice, potentially creating new avenues for the treatment of liver damage in schistosomiasis.

## Background

Schistosomiasis is a widespread parasitic disease and affects more than 200 million people in many developing countries in tropical and subtropical areas [1]. The schistosome egg-induced hepatic granulomatous response, which subsequently results in serious liver fibrosis, is central to the morbidity and mortality in patients with schistosomiasis [2]. Thus, the effective suppression of granulomatous response-trigger liver fibrosis is crucial for schistosomiasis treatment.

Hepatic fibrosis is the process of excessive deposition of collagenous and noncollagenous extracellular matrix components in the liver [3]. Indeed, activated HSCs are responsible for this process during hepatic fibrogenesis [4]. Prevention of HSC activation has been viewed as a potential therapeutic strategy for liver fibrosis [5]. Although some potential anti-fibrotic targets have been identified, effective clinical therapies are still lacking. Therefore, it is important to develop novel strategies to suppress HSC activation for the treatment of fibrotic hepatic diseases, such as schistosomiasis.

Mesenchymal stem cells (MSCs) represent a promising therapeutic strategy for schistosome-induced liver injury [6–10], but this approach has been hindered by a limited understanding of the MSC-mediated control of liver injury in schistosomiasis. Extracellular vesicles (EVs) are small membrane vesicles released by almost all cell types, which contribute to donor cell-mediated biological effects [11, 12]. Indeed, EVs contain a subset of proteins, lipids, and nucleic acids derived from the donor cell and play important roles in intercellular communication through transferring their contents, including proteins, lipids, and RNAs, between cells [13]. EVs produced by MSCs (MSC-EVs) exert therapeutic effects in several disease models such as chemotherapy-induced mouse model of premature ovarian failure [14], experimental allergic asthma [15], and *Escherichia coli* endotoxin-induced acute lung injury [16]. In addition, EVs have reduced potential side effects because they are less complex and better defined as compared to MSC therapies, and there are additional logistical advantages of using EVs, which can be considered as an off-the-shelf product. Thus, MSC-EVs may be a promising alternative to the MSC therapy for various types of disease [17]. However, the role of MSC-EVs in schistosomiasis remains unclear.

In this study, we demonstrated that similar with the hUCMSCs, hUCMSC-EVs also have the capacity to alleviate *S. japonicum*-induced liver damage. More importantly, hUCMSC-EV-mediated amelioration of liver injury concomitant with decreased expression of α-smooth muscle actin (α-SMA), collagen 1, and collagen 3 and, as a consequence, increased the survival of schistosome-infected mice. Mechanistically, hUCMSC-EVs inhibited pro-fibrogenic responses of HSC such as viability and proliferation and decreased its expression of pro-fibrogenic markers α-SMA, collagen 1, and collagen 3 stimulated by TGF-β1 in vitro. Our findings indicated that hUCMSC-EVs suppress hepatic stellate cell proliferation and activation and improve liver injury in schistosomiasis.

## Materials and methods

### Generation of hUCMSCs and hUCMSC-EVs

Human umbilical cord samples were obtained from informed, consenting mothers at the First People’s Hospital of Nanjing (Nanjing, China) and the procedures of isolation and characterization of human umbilical cord MSCs (hUCMSCs) were performed as our previously described method [18]. hUCMSC-EVs were prepared from the supernatant of hUCMSCs by ultracentrifugation (Beckman Coulter Optima L-100 XP ultracentrifuge, Miami, FL). First, hUCMSCs were cultured approximately 80% confluent in 10 cm culture dishes. The medium was replaced with serum-free culture medium, and the cells were cultured for 24 h, followed by the collection of hUCMSC culture supernatants and centrifugation at 300×*g* for 10 min and 2000×*g* for 20 min. After that, the supernatants were collected and subjected to ultracentrifugation at 100,000×*g* for 90 min at 4 °C. The pellets were gathered and resuspensed in PBS, subjected to a second ultracentrifugation. Next, the particle size distribution and the quantification of the hUCMSC-EV number were measured by nanoparticle trafficking analysis using NanoSight NS300 (Malvern Instruments Ltd., Worcestershire, UK) according to the manufacturer’s protocol. The hUCMSC-EVs aliquots were stored at − 80 °C until needed.

### Transmission electron microscopy

The purified hUCMSC-EVs were resuspended in PBS and dropped onto carbon-coated electron microscope grids. After incubation at ambient temperature for 5 min, the lattice was negatively stained with 2% phosphotungstic acid solution for 30 s. Then, the grids were examined and photographed using a transmission electron microscope (JEM-1200EX; JEOL Ltd., Tokyo, Japan).

### Flow cytometer

The purified hUCMSC-EVs were resuspended in PBS, stained with 5 μL of the directly fluorescent antibody CD90 and CD73 (BD Biosciences, San Jose, CA) respectively for the detection of the typical surface markers of MSC. Non-stained EVs were used as control, and the phenotype of hUCMSC-EVs was analyzed by using BD Accuri C6 Flow Cytometer (BD Biosciences).

### Parasites and animals

Cercariae of *S. japonicum* were obtained from *Oncomelania hupensis* snails, which were purchased from the Jiangsu Institute of Parasitic Diseases (Wuxi, China).

Six-week-old male BABL/c mice were purchased from the Laboratory Animal Center of Nanjing Medical University (Nangjing, China). For infections, mice were infected percutaneously with 14–26 *S. japonicum* cercariae.

### Grouping and treatment information

To determine the therapeutic potential of hUCMSC-EVs compared to the cells themselves, according to previous reports [16] and our previous study [18], 7.5 × 10^5^ hUCMSCs/3 × 10^9^ EVs or 5 × 10^5^ hUCMSCs/2 × 10^9^ EVs were injected simultaneously into *S. japonicum*-infected mice. The specific grouping and treatment information are as follows:

For survival analysis, 32 mice were randomly divided into four groups (control, PBS, MSCs, and EVs). Mice in control group were left uninfected and mice in groups PBS, MSC, and EVs were infected with 26 cercarie. Mice in group PBS served as infected controls which is treated by PBS (once a week, for four consecutive weeks, 0.2 mL; intravenous); mice in MSC group were injected with hUCMSCs (7.5 × 10^5^ cells suspended in 0.2 mL PBS; intravenous); mice in EV group were injected with hUCMSC-EVs (3 × 10^9^ suspended in 0.2 mL PBS; intravenous).

Thirty-two mice (one of the three independent experiments; 96 mice in total three experiments) were randomly divided into four groups (control, PBS, MSCs, and EVs). Mice in the control group were uninfected. In groups of PBS, MSCs, and EVs, each mouse was infected with 14 cercarie, and at the fourth week after infection, mice in these groups were injected with 0.2 mL PBS, hUCMSCs (5 × 10^5^ cells suspended in 0.2 mL PBS; intravenous) or hUCMSC-EVs (2 × 10^9^ suspended in 0.2 mL PBS; intravenous) respectively. Mice were sacrificed by anesthesia overdose at 2 (42 days) or 4 weeks (56 days) after consecutive treatment (once a week).

Twenty-four mice (one of the three independent experiments; 72 mice in total) were randomly divided into four groups (Control, PBS, MSCs, and EVs). Mice in the control group were uninfected. In groups PBS, MSCs, and EVs, each mouse was infected with 20 cercarie, and at the sixth week after infection, mice in these groups were treatment by praziquantel (PZQ) (500 mg/kg; intragastric). Then, mice in the PBS, MSCs, and EVs groups were injected with either 0.2 mL of PBS (intravenous), 0.2 mL of hUCMSCs (5 × 10^5^ cells suspended in PBS; intravenous), or 0.2 mL of hUCMSC-EVs (2 × 10^9^ suspended in PBS; intravenous). Mice were sacrificed by anesthesia overdose at 70 days.

### Liver histopathology and fibrosis measurement

Liver tissues were fixed in 10% neutral buffered formalin. Paraffin embedded sections were dewaxed and stained with hematoxylin and eosin (H&E) for granulomas analysis or Masson’s trichrome staining for fibrosis analysis. All pictures were captured using an Axiovert 200 M microscope (Carl Zeiss GmbH, Jena, Germany), and granulomas were analyzed using AxioVision Rel 4.7 (Carl Zeiss). Fibrosis was detected histologically by measuring the intensity of fibrosis in six random digital images captured from collagen-specific blue-stained slides of each mouse using Image-Pro Plus software (Version X; Adobe, San Jose, CA). The mean optical density of collagen was determined by dividing integral optical density by the image area.

The collagen content of the liver, determined as hydroxyproline content, was measured using a colourmetric assay according to manufacturer’s protocol (Nanjing jiancheng Bioengineering Institute, Nanjing, China).

After the liver tissue was digested by 4% KOH, the number of *S. japonicum* eggs in the liver was measured.

### Detection of ALT, AST and endotoxin in serum

Levels of serum alanine transaminase (ALT) and aspartate aminotransferase (AST) were assayed using an Olympus AU2700 Chemical Analyzer (Olympus, Tokyo, Japan).

Levels of endotoxin in serum were measured using commercial enzyme-linked immune sorbent assay according to manufacturer’s instructions (Xiamen Bioendo Technology Co., Ltd., Xiamen, China).

### hUCMSC-EVs labeling and tracking in mice

hUCMSC-EVs were labeled with Dir (5 μg/mL; Invitrogen, Carlsbad, CA) and incubated under 37 °C conditions for 30 min. Then, the Dir-labeled EVs were washed twice with PBS using unltracentrifugation method. For tracking the distribution of hUCMSC-EVs in *S. japonicum*-infected mice, 3 × 10^9^ Dir-labeled hUCMSC-EVs were injected into *S. japonicum*-infected mice (28 day post-infection) and fluorescence intensity were detected using an IVIS Spectrum (PerkinElmer, Waltham, MA). After hUCMSC-EV injection via the tail vein, scans were performed at 630 nm. In vivo spectral imaging from 690 to 850 nm was measured using an exposure time of 150 ms per image frame.

### Immunohistochemisty

Briefly, liver tissue slides were incubated with monoclonal primary antibodies against α-SMA (1:200; Abcam, Cambridge, MA) at 37 °C for 90 min, followed by washing and incubation with HRP-conjugated secondary antibody (1:2000; Abcam) at 37 °C for 20 min. Then, slices were visualized with 3, 30-diaminobenzidine and counterstained with Harris’s hematoxylin for microscopic examination.

### Western blotting

The hUCMSC-EVs, hUCMSCs, or liver tissues were collected in RIPA buffer (Cell Signaling Technology Inc., Danvers, MA) containing PMSF (Beyotime, Nantong, China) and quantified using a BCA Protein Assay Kit (Beyotime). Equal amounts of proteins (50 μg) were electrophoresed in 10% sodium dodecyl SDS-PAGE and transferred onto polyvinylidene difluoride membranes (Bio-Rad, Hercules, CA), as our previous description [19]. The antibodies to human CD63, calreticulin, α-SMA, β-actin, and HRP-linked anti-rabbit IgG were all purchased from Abcam.

### Cell culture

The human hepatic stellate cell line LX-2 was purchased from ATCC (Manassas, VA). LX-2 cell was not revalidated for this work and was grown in DMEM medium (Gibco, Carlsbad, CA) supplemented with 10% fetal bovine serum (Gibco) and 1% pen/strep (Gibco) under 5% CO_2_ and 37 °C conditions.

### RNA isolation and quantitative real-time PCR

RNA was extracted from the liver tissues or LX-2 cell treated by hUCMSC-EVs using Trizol (Invitrogen) according to the manufacturer’s protocol. The real-time PCR primers of α-SMA, collagen 1, collagen 3, IL-1β, TNF-α, INF-γ, and GAPDH were purchased from Genecopoeia (Germantown, MD). Real-time PCR was performed with All-in-one™ qPCR Mix (Genecopoeia) in a CFX96™ Real-Time system (Bio-Rad, Hercules, CA). The relative expression of mRNA was evaluated by the 2^−ΔΔCt^ method and normalized to GAPDH, based on our previous description [19].

### Cell viability assay and cell proliferation

The cell viability was analyzed using the cell counting kit-8 (KeyGEN BioTECH, Nanjing, China). LX-2 cells were seeded in 96-well plates (5 × 10^3^ cells per well) and cultured overnight. For the cell viability assay, the medium was replaced with 100 μL of EV-free completed medium (DMEM medium supplemented with EV-free serum and 1% pen/strep) in the absence or presence of various concentrations of the hUCMSC-EVs for 24 or 48 h. Then, 10 μL of CCK-8 was added to each well and the cells were incubated for 1.5 h. Finally, the absorbance of the cells in each well was determined at 450 nm using a microplate reader (Synergy HT, BioTek, Biotek Winooski, VT). Culture medium without cells was used as blank control.

Cell proliferation was measured using an iClick™ EdU Assay Kit (Genecopoeia), according to the manufacturer’s instructions.

### Statistical analysis

The statistical analyses were performed with GraphPad Prism (Version 5.0; La Jolla, CA). The data are expressed as the means ± SD. Statistical significance was determined using the Kaplan-Meier method, one-way analysis of variance. Data were considered statistically significant for *P* values less than 0.05.

## Results

### Identification of human umbilical cord mesenchymal stem cells (hUCMSCs) and hUCMSC-extracellular vesicles (hUCMSC-EVs)

**Fig. 1:**
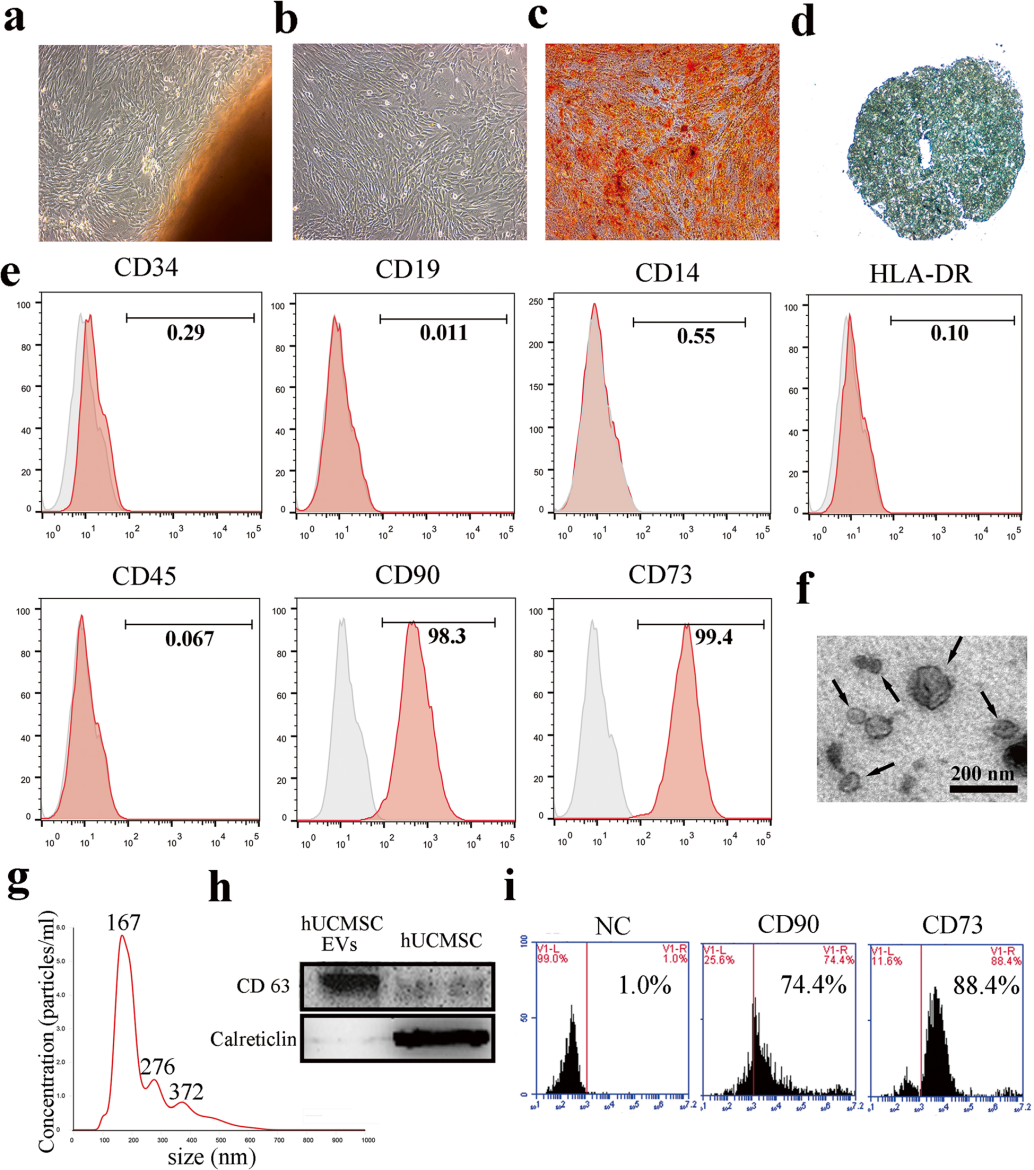
Characteristics of hUCMSCs and hUCMSC-EVs. Morphology of **a** passage 0 and **b** passage 3 hUCMSCs were observed under light microscopy (× 100). Representative images of osteocyte (× 100) and chondrocyte (× 200) differentiation of hUCMSCs cultured in the differentiation medium. The cells were stained with **c** Alizarin Red and **d** Alcian Blue. **e** Results for the flow cytometry analyses of phenotypic markers related to MSCs. **f** hUCMSC-EVs were observed under a transmission electron microscope; some of the hUCMSC-EVs are indicated by arrows. Scale bar = 200 nm. **g** Size distributions of hUCMSC-EVs were detected using the NTA. **h** Western blotting analysis of CD63 and Calreticlin expression in lysates from hUCMSC-EVs and hUCMSCs. **i** Flow cytometry detection of CD73 and CD90. EVs reacted with the isotype antibody were applied as the negative control (NC)

We firstly isolated and characterized the hUCMSCs. The results showed that the cells displayed long spindle-like shapes, formed colonies, and reached confluency (Fig. 1a, b). Furthermore, these cells had multi-lineage potential to differentiate into osteocytes and chondrocyte (Fig. 1c, d). Importantly, the immunophenotype of these cells were monitored by flow cytometry and revealed that these cells were positive for CD90 and CD73, but negative for CD34, CD19, CD14, HLA-DR, and CD45 (Fig. 1e). Taken together, these results demonstrated that we had efficiently generated hUCMSCs, as confirmed on the basis of the criteria defined by the International Society for Cellular Therapy [20].

In addition, we purified and characterized the hUCMSC-EVs. The data showed that hUCMSC-EVs were round membrane-bound vesicles (Fig. 1f) and have an average diameter of about 170 nm with a size distribution of 50 to 650 nm (Fig. 1g). Western blotting demonstrated that the EV marker proteins CD63 were present in these vesicles (Fig. 1h). In addition, we also found that hUCMSC-EVs expressed the MSC-specific markers CD90 and CD73 (Fig. 1i).

### hUCMSC-EVs prolong the survival of *S. japonicum*-infected mice

**Fig. 2:**
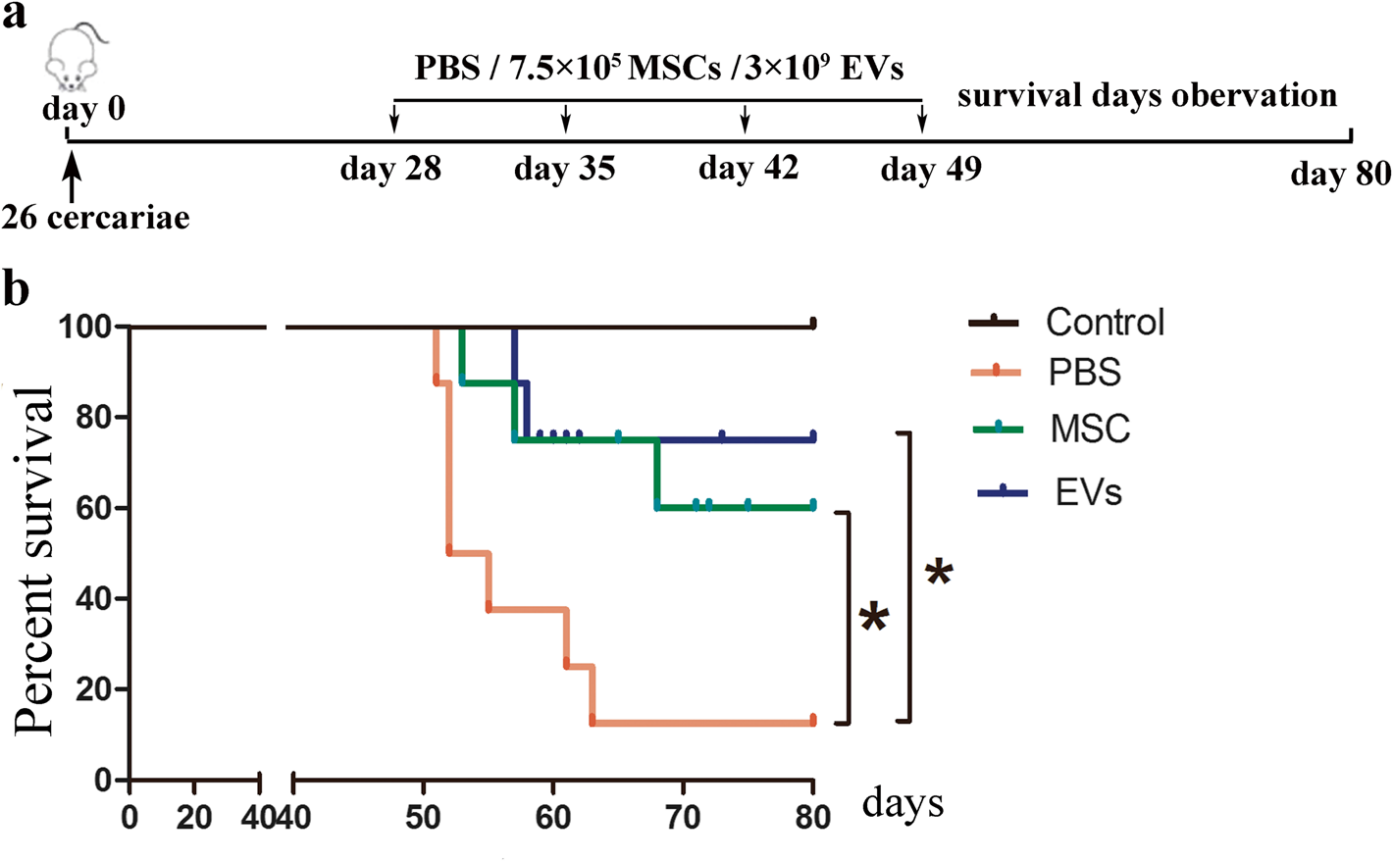
hUCMSC-EVs prolong the survival of *S. japonicum*-infected mice. **a** Time schedule for parasite infection and injections of PBS, hUCMSCs, or hUCMSC-EVs. Mice were infected percutaneously with 26 *S. japonicum* cercariae at day 0 and treated with hUCMSCs/hUCMSC-EVs by tail vein injection at day 28/35/42/49 post-infection. The animals were subjected to an 80-day survival study. MSCs, hUCMSCs; EVs, hUCMSC-EVs. **b** The survival curves of mice in each group. *P < 0.05, compared with the PBS group

Firstly, in order to investigate the efficacy of hUCMSC-EVs in final survival, mice were infected with 26 parasites that would lead to severer liver damage of the mice. Based on the report of Haldar et al. [21], more hUCMSCs (total 3 × 10^6^) and relative hUCMSC-EVs were injected (Fig. 2a). We found that hUCMSC administration significantly increased the survival of mice with schistosomiasis japonica, more importantly, hUCMSC-EVs showed a similar effection with the hUCMSC treatment (Fig. 2b).

### hUCMSC-EVs attenuate the progression of liver pathology in *S. japonicum*-infected mice

**Fig. 3:**
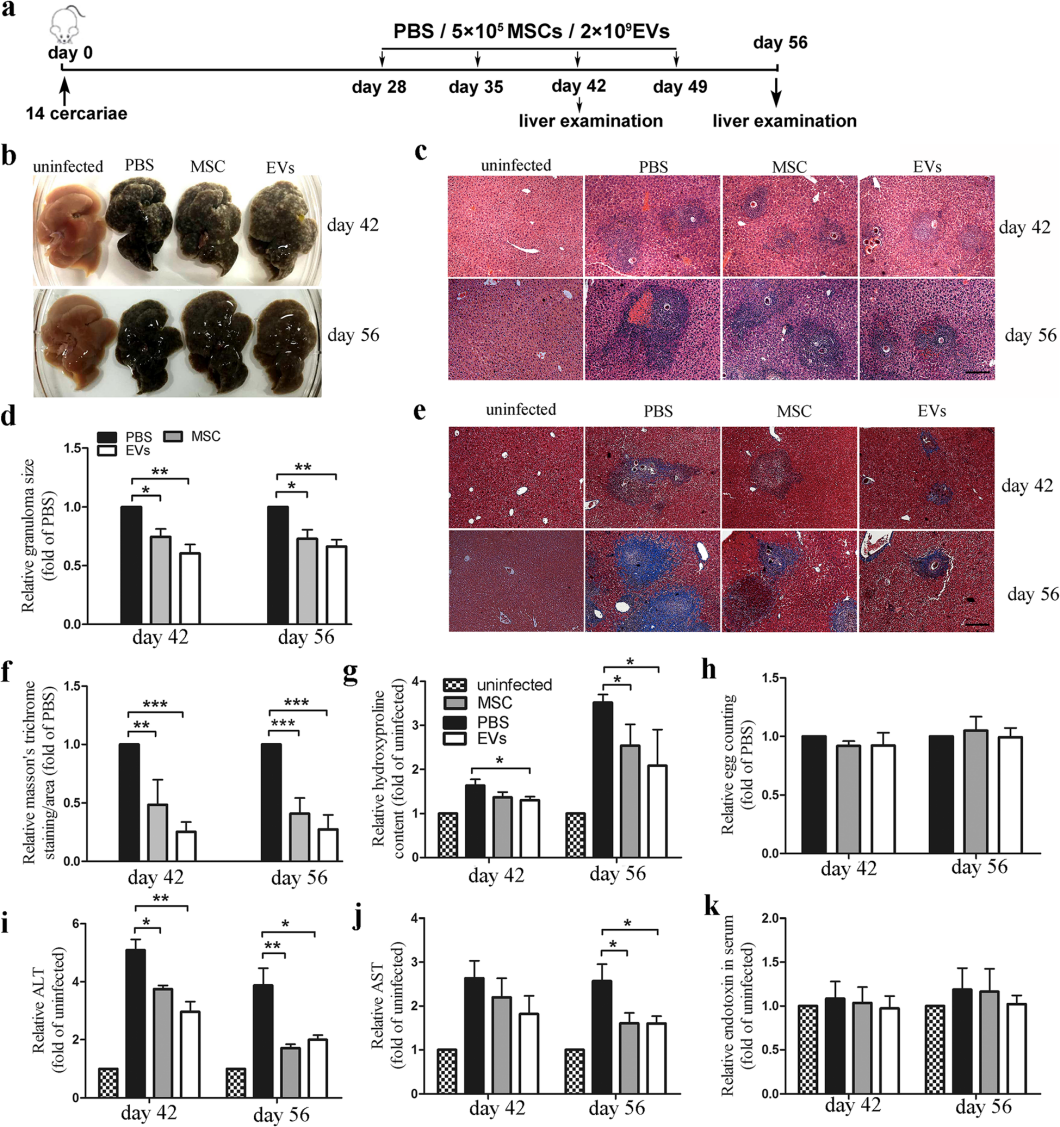
hUCMSC-EVs attenuated *S. japonicum*-induced liver injury in mice. **a** Time schedule for parasite infection and intravenous injection of hUCMSCs, hUCMSC-EVs, or PBS. Mice were infected percutaneously with 14 *S. japonicum* cercariae at day 0 and received hUCMSC, hUCMSC-EVs, or PBS by tail injection at day 28/35/42/49 post-infection. Liver samples and blood serum were collected at the indicated time points. MSCs, hUCMSCs; EVs, hUCMSC-EVs. **b** The appearance of liver from each group at the indicated time points. **c** HE staining of liver sections. Scale bar = 200 μm. **d** Mean area of granuloma measured from HE staining liver sections using a calibrated measuring eyepiece. Striped bars, uninfected mice; black bar, PBS control; gray bars, hUCMSC group; white bars, hUCMSC-EVs group. **e** Masson’s trichrome staining of collagen in the liver sections. Scale bar = 200 μm. **f** Fibrosis scores measured from Masson’s trichrome staining liver sections. **g** Collagen content of the livers determined as hydroxyproline content. **h** The number of schistosome eggs in the liver was measured after the liver tissue was digested by 4% KOH. Levels of **i** ALT, **j** AST, and **k** endotoxin in blood serum. Mean values ± SD for three independent experiments are shown, *P < 0.05; **P < 0.01; ***P < 0.001, compared with the PBS group

Given that the schistosome egg-induced liver damage is central to the mortality in the host infected with schistosome, we investigated whether hUCMSC-EVs could ameliorate liver injury during schistosome infection. To this end, mice were exposed to 14 parasites that would lead to a slower disease progression, and less dosage of hUCMSCs (total 2 × 10^6^) or hUCMSC-EVs was used (Fig. 3a). The administration of hUCMSC-EVs, as well as hUCMSCs, significantly decreased the formation of hepatic granulomas at 42 or 56 day after infection (Fig. 3b–d). Masson’s trichrome staining of liver sections showed significantly reduced collagen deposition and hydroxyproline content in mice treated with hUCMSC-EVs or hUCMSCs, compared with control groups (Fig. 3e–g). There were no significant differences of the egg burden among these groups (Fig. 3h). Furthermore, the circulating concentration of alanine transaminase (ALT) and aspartate transaminase (AST) was significantly lower in hUCMSC-EV- or hUCMSC-treated mice than that in PBS-treated mice, while the concentration of serum endotoxin was similar in all groups (Fig. 3i–k). Taken together, these results suggested that the hUCMSC or hUCMSC-EV-mediated amelioration of liver injury may contribute to increased survival of mice treated with hUCMSC-EVs or hUCMSCs.

### Therapeutic potential of hUCMSC-EVs in liver fibrosis in schistosome-infected mice after praziquantel therapy

**Fig. 4:**
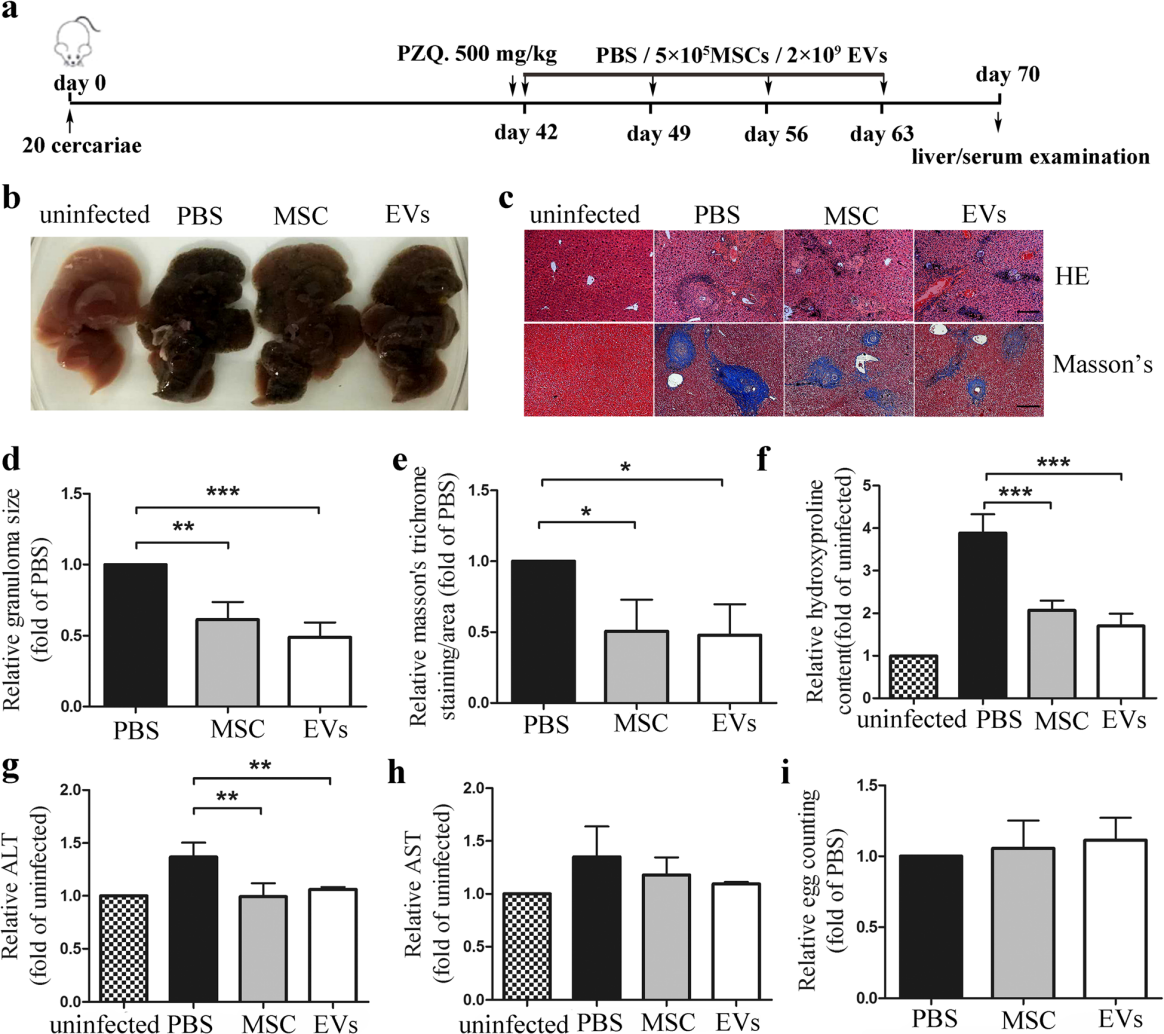
The therapeutical effect of hUCMSC-EVs to *S. japonicum*-mediated hepatic fibrosis after praziquantel therapy. **a** Time schedule for parasite infection and intravenous injections of PBS, hUCMSCs, or hUCMSC-EVs. Mice were infected percutaneously with 20 *S. japonicum* cercariae. The infected mice were treated with praziquantel (PZQ) to kill the adult worms, and then received hUCMSCs, hUCMSC-EVs, or PBS by tail infection at day 42/49/56/63 post-infection. Liver and serum samples of the mice were collected at day 70 post-infection. MSCs, hUCMSCs; EVs, hUCMSC-EVs. **b** The appearance of liver from each group. **c** HE and Masson’s trichrome staining of liver sections. Scale bar = 200 μm. **d** Mean area of granuloma determined from HE staining liver sections. **e** Fibrosis score measured from Masson’s trichrome staining liver sections. **f** Collagen content of the livers was detected as hydroxyproline content. Levels of **g** ALT and **h** AST in serum. **i** The number of schistosome eggs in the liver was measured after the liver tissue was digested by 4% KOH. Mean values ± SD for three independent experiments are shown, *P < 0.05; **P < 0.01; ***P < 0.001, compared with the PBS group

Praziquantel (PZQ) is currently the available safest, cheapest, and most efficient anti-schistosomal drug [22]. However, the continuous progression of liver damage, especially liver fibrosis, is not self-limiting in patients after PZQ [23]. On 28 days post-infection, immature eggs start to be produced by adult worms, which need extra 7–14 days to develop into mature miracidia to secret soluble egg antigens and toxins to cause liver pathology. PZQ chemotherapy at 42 days is a simulated clinical treatment on patients. PZQ only kills adult worms but has no effect on miracidia in eggs. Thus, in order to investigate whether hUCMSC-EVs have therapeutic potential in liver injury after infection, mice were treated with PZQ at 42 days post-infection to kill adult worms to avoid the continues laying eggs and over-serious liver pathology (Fig. 4a). Results showed that the area of granuloma and the severity of liver fibrosis, but not egg burdens, were significantly reduced in infected mice treated with hUCMSC-EVs as well as hUCMSCs, concomitant with decreased levels of ALT in serum (Fig. 4b–i). Taken together, these results indicated that the hUCMSC-EVs also have the ability to inhibit the progression of liver injury in mice after treatment with PZQ therapy.

### hUCMSC-EVs inhibit the activation of hepatic stellate cells during schistosome infection

**Fig. 5:**
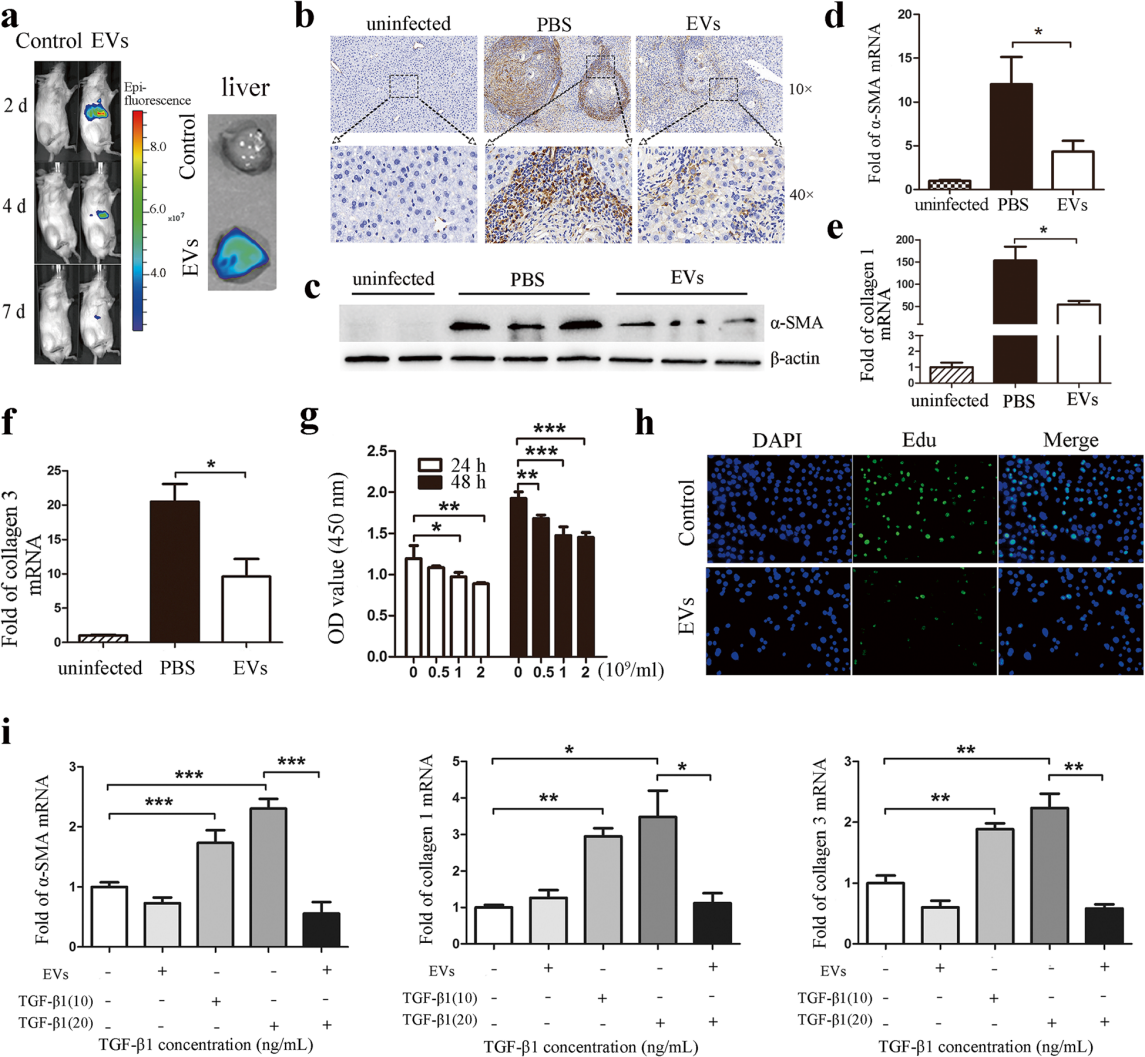
hUCMSC-EVs inhibited the activation of HSCs. **a** Distribution of DIR-labeled hUCMSC-EVs in *S. japonicum*-infected mice (left, 28-day infection) and in infected mice liver (right, DiR-labeled EVs treatment 2 days) after tail vein administration. Control, uninfected mice injected with PBS; EVs, infected mice administrated DiR-labeled hUCMSC-EVs. **b** Immunohistochemical staining and **c** western blotting results of α-SMA in liver sections from infected mice after 56-day infection. EVs, hUCMSC-EVs. Real-time PCR analysis of **d** α-SMA, **e** collagen 1, and **f** collagen 3 in the liver samples from infected mice after 56-day infection. *P < 0.05, compared with the PBS group. **g** LX-2 cells (5 × 10^3^) were incubated in the presence or absence of hUCMSC-EVs for 24 h or 48 h. The cell viability was determined via the CCK8 assay. *P < 0.05; **P < 0.01; ***P < 0.001, compared with the 0 group. **h** LX-2 cells (5 × 10^3^) were incubated in the presence or absence of hUCMSC-EVs (2 × 10^9^/ml) for 48 h. The cell proliferation was measured using EdU cell proliferation assay. Real-time PCR analysis for TGF-β1-induced or hUCMSC-EVs reversed fibrosis-related **i** α-SMA, collagen 1 and collagen 3 products. *P < 0.05; **P < 0.01; ***P < 0.001, compared with the control or TGF-β1 treatment (20 ng/mL) group

To further explore the mechanisms of hUCMSC-EV-mediated suppression of liver injury in schistosomiasis, we first used DiR-labeled EVs to detect the distribution of hUCMSC-EVs in infected mice. The result showed that DiR-EVs were mainly restricted to the liver region, and the content gradually decreased in a time-dependent manner (Fig. 5a). The expression of α-SMA was significantly reduced in the liver of the hUCMSC-EV group compared to PBS group (Fig. 5b–d). We also analyzed the relative expression of collagen 1 and collagen 3 in liver by real-time PCR and found that the collagen 1 and collagen 3 expression level was significantly downregulated in hUCMSC-EV group (Fig. 5e, f).

In vitro experiments, we found that hUCMSC-EVs directly inhibit LX-2 cell (immortalized human HSCs) viability and proliferation (Fig. 5g, h). Furthermore, the addition of hUCMSC-EVs significantly inhibited the expression of α-SMA, collagen 1 and 3 in LX-2 stimulated with TGF-β1 (Fig. 5i). Thus, these results suggested that hUCMSC-EVs suppress hepatic stellate cell activation induced by TGF-β1 and ameliorate liver injury in schistosomiasis.

### hUCMSC-EV treatment downregulates liver inflammation during schistosome infection mice

**Fig. 6:**
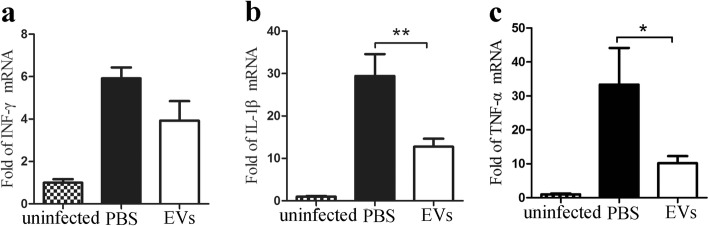
hUCMSC-EV treatment downregulates liver inflammation in schistosome infection mice. Real-time PCR analysis of **a** INF-γ, **b** IL-1β, and **c** TNF-α in the liver samples from infected mice after 56-day infection. *P < 0.05; **P < 0.01, compared with the PBS group

Inflammatory response is an important player in liver fibrosis development. Therefore, the inflammation in the liver of the hUCMSC-EV or PBS groups was investigated. Analysis of cytokines’ mRNA levels revealed a non-statistically significant decrease in INF-γ but significantly lower levels of TNF-α and IL-1β after hUCMSC-EV treatment (Fig. 6a–c), suggesting that hUCMSC-EVs inhibited inflammatory responses in schistosome-infected mice livers.

## Discussion

Schistosoma infection is a widespread parasitic infectious disease and the liver injury induced by parasite egg is still a challenge for clinical treatment [1]. MSC transplantation is thought to be quite effective [6, 7, 24, 25]; however, this approach has been hindered by the concerns that MSCs might trigger pulmonary embolism in some clinical settings [26]. In the present study, we found that like hUCMSCs, EVs derived from hUCMSCs have also the capacity to ameliorate *S. japonicum*-induced liver damage and increased survival of schistosome-infected mice. These findings not only support the notion that MSC-EVs are a major kind of functional forms of MSC [27] but also suggest that MSC-EVs may be a promising alternative to MSC therapy for schistosomiasis.

Mortality from schistosomiasis japonica and mansoni is mainly associated with hepatic histopathology [28]; however, the effective intervention strategies are still lacking. Although one report indicated that the administration of hUCMSCs is associated with decreased liver damage in mice infected with *S. mansoni* [7], it still remains unclear the effect of hUCMSCs on the outcome of schistosomiasis, especially schistosomiasis japonica, a parasitic disease with more egg deposition in host liver and more severe liver damage. In this study, we are the first to report that hUCMSC treatment increases the survival of *S. japonicum*-infected mice. Moreover, hUCMSC-EVs exhibited the similar effects to hUCMSCs. Although we could not fully explain whether hUCMSC-mediated increase in the survival of *S. japonicum*-infected mice was solely through the release of EVs, hUCMSC-EVs may be an effective intervention strategy for treating schistosomiasis disease.

In recent years, evidences support that MSC-derived EVs play therapeutic roles in several kinds of liver damage induced by CCL4 [29], D-GalN/LPS [30], hepatic ischemia reperfusion [31], and hepatic S100 [32]. Our data further showed that similar with hUCMSC treatment, hUCMSC-EV injection also efficiently alleviate the liver granuloma and fibrosis in schistosome-infected mice, with concomitant decreased levels of ALT and AST in serum, which may contribute to increased survival of schistosome-infected mice treated with hUCMSC-EVs.

Accumulating evidence has revealed that MSC-EVs may retain the homing properties of their parent cells to injured tissues [33]. Our group previously reported that hUCMSC-EVs aggregate to nerve defects in a rat model of sciatic nerve transaction [34]. Similar with these published literature, our data also show that hUCMSC-EVs are able to inhabit the site of inflammation in infected mice. Although the precise molecular mechanisms by which hUCMSC-EVs migrate into sites of injury remain to be defined, these findings suggest that hUCMSC-EVs are capable of entering into and performing functions within injured livers.

HSCs are the intermediate bridge between hepatic inflammation and hepatic fibrosis in fibrotic liver disease [35]. Although we did not rule out the possibility that it might be the direct protective effect of hUCMSC-EVs on liver cells or the indirect effects target on the other cells, e.g., macrophages or T cells, our data suggested that hUCMSC-EV-meditated suppression of TGF-β1-induced HSC activation may be involved in the amelioration of liver fibrosis in schistosomiasis. It was reported recently that the administration of human-induced pluripotent stem cell-derived EVs reduces development of fibrosis and TGF-β1-induced HSC activation in an experimental model of cholestatic liver fibrosis [36]. In contrast, some published studies have indicated that adipose MSC-EVs (AMSC-EVs) had no demonstrable effect on the proliferation or activation of HSCs [37, 38]. The different efficacy of these EVs could be due to the different source of MSCs. The exact mechanism of how hUCMSC-EVs inhibit the activation of HSCs needs to be investigated in the future study.

## Conclusions

Our results demonstrated that hUCMSC-EV control of HSC proliferation and TGF-β1-induced activation may contribute to its mediated amelioration of liver injury in the context of schistosome infection. hUCMSC-EVs may be a better therapeutic option for liver injury in schistosomiasis.

## Data Availability

All data generated or analyzed during this study are included in this published article.

## Abbreviations

EVs: Extracellular vesicles
HSC: Hepatic stellate cell
hUCMSCs: Human umbilical cord MSCs
IL-1β: Interleukin-1 beta
INF-γ: Interferon-γ
MSCs: Mesenchymal stem cells
PZQ: Praziquantel
*S. japonicum*: *Schistosoma japonicum*
TGF-β1: Transforming growth factor-β1
TNF-α: Tumor necrosis factor alpha
α-SMA: α-smooth muscle actin
